# Navigating TAM receptor dynamics in tumour immunotherapy

**DOI:** 10.1007/s00262-024-03879-z

**Published:** 2025-03-15

**Authors:** Jihao Yang, Guanmin Chen, Rui Wang, Chengcheng Song, Huaqiang Yi

**Affiliations:** School of Acupuncture and Tuina, Shandong University of Traditional Chinese, Medicine, Jinan, 250013 People’s Republic of China

**Keywords:** TAM receptor, TYRO3, AXL, MERTK, Tumour

## Abstract

The TAM receptor family is getting more and more attention in the field of tumour immunity. Activation of TAM receptors not only aids in the survival and multiplication of tumour cells but also increases their likelihood of invading other cells and spreading. In addition, activation of TAM receptors helps to inhibit the anti-tumour immune response, allowing tumour cells to evade immune surveillance. In terms of therapeutic strategies, a number of inhibitors targeting TAM receptors are in preclinical and clinical development. Despite significant progress in clinical trials in recent years, challenges remain. This review delves into the kinetic characteristics of the TAM receptor family, their dual role in tumour immunity, and the transmission process of downstream signalling pathways. Based on this, we analysed and summarised the unique strategies and combination therapies for regulating tumour immunity using TAM receptor inhibitors. It not only helps to elucidate the key role of TAM receptors in tumour immunity but also provides new perspectives and strategies for future tumour therapy.

**Supplementary Information:**

The online version contains supplementary material available at 10.1007/s00262-024-03879-z.

## Introduction

Tumour immunotherapy is a groundbreaking approach to treating tumours that aims to activate the patient's immune system, so it can recognise and eliminate tumour cells. Human malignancies have widely documented the overexpression or aberrant activation of TAM receptors [1, 2], highlighting their importance in tumour growth. TYRO3, AXL, and MERTK are three receptors that belong to the TAM family of tyrosine kinase receptors. These receptors play a crucial role in regulating the immune system, eliminating dead cells, facilitating tissue regeneration, and impacting tumour formation. Some ligands, including growth arrest specific protein 6 (Gas6) and protein S (Pros1), have been identified [3, 4]. These ligands facilitate the ability of TAM receptors to enhance macrophage phagocytosis. They accomplish this by identifying Phosphatidylserine (PtdSer) on the outer layer of cells undergoing apoptosis or experiencing stress [5]. This process is essential for preserving the equilibrium of the immune system and averting autoimmune reactions [6–8]. The activation of TAM receptors results in the suppression of proinflammatory cytokines and the elevation of immunosuppressive cytokines, thereby establishing an immunosuppressive tumour microenvironment (TME) [9, 10]. TAM receptors have a role in regulating the function and growth of immune cells, affecting T lymphocytes (T cells), NK cells, DCs, and other types of cells. They also help in promoting the activity and/or recruitment of cell populations that inhibit the immune system [11–13]. TAM receptors may stop the start of immune responses by releasing chemicals that help repair tissue [14]. This facilitates the development of a tumour exhibiting traits akin to M2 macrophages, thereby establishing an environment that hampers the immune system in its vicinity.

TAM receptors play a twofold role in the TME. They have the ability to either facilitate the proliferation and progression of malignancies or impede tumour growth in specific instances [15]. Macrophages and dendritic cells (DCs) specifically exhibit this phenomenon [16]. TAM receptors are associated with the polarisation of tumour-infiltrating macrophages [17–19]. It is very important for macrophages to be able to get rid of dying cells [20]. AXL can stop the proinflammatory M1 macrophage state, stop antigen-presenting cells from blocking Toll-like receptor (TLR) responses, and improve the function of natural killer (NK) cells [15, 21]. TAM receptors expressed on DCs contribute to the attenuation of the inflammatory response by exerting negative regulatory effects on TLR signalling. The upregulation of SOCS1/SOCS3 facilitates this [22].

The results highlight the complex function of TAM receptors in the tumour immune system. Our research has discovered that TAM receptors and their ligands provide promising prospects for investigating the application of immune checkpoint blockades in tumour therapy. Therefore, it is crucial to have a comprehensive understanding of the function of TAM receptors in the immune environments of tumours in order to create effective therapeutic approaches. This paper provides a concise overview of recent findings concerning the involvement of the TAM receptor family in tumour immunotherapy. The analysis delved deeply into the complex involvement of patients and emphasised the need to develop new treatment protocols and personalised treatment plans. Furthermore, combined treatment strategies may provide broader development opportunities for the regulation of tumour immunity.

## Structural features of the TAM receptor family and its ligands

The TAM receptor family is one of the 20 subfamilies of receptor tyrosine kinases (RTKs) on the cell surface, consisting of three members: TYRO3, AXL, and MERTK (Fig. S1**)**. These receptors have significant structural similarities, including two Ig-like domains, two fibronectin type III (FN III) domains, one hydrophobic transmembrane domain, and one tyrosine kinase domain. The two Ig-like domains at the N-terminus of the receptor are involved in binding to the ligand, while the two FNIII regions help to strengthen the interaction between the receptor and the ligand. The tyrosine kinase domain is located at the C-terminus of the receptor. When the receptor interacts with its ligand, the tyrosine residues within this domain are phosphorylated, activating downstream signalling pathways. TAM family receptors have a single hydrophobic transmembrane domain, consisting of an extracellular ligand-binding domain of neural cell adhesion molecules, a transmembrane region, and an intracellular region with a tyrosine kinase domain. Therefore, they possess characteristics of adhesion molecules as well as the activity of tyrosine kinases. Compared to TYRO3, AXL and MERTK receptors have a higher structural similarity, with 31 to 36% of homologous amino acids in the extracellular region and 54 to 59% in the intracellular region [23]. TYRO3, AXL, and MERTK are homologous type I RTKs that share a conserved sequence [KW(I/L) and (I/L)ES] in their kinase domains and have similar extracellular structural organisation. The two Ig-like domains and two FN III repeat sequences in the extracellular region can bind to ligands; the intracellular region contains a tyrosine protein kinase domain, which has tyrosine protein kinase activity and phosphorylation sites, playing a role in the transmembrane transduction of cellular signals. The DNA sequences of the TYRO3, AXL, and MERTK genes are very similar, each being 3–5 kb in size. TYRO3 and AXL contain 20 exons, while MERTK contains 19 exons [23–25] The ligands Gas6 and ProS 1 are both members of the vitamin K-dependent protein family, with 42% homologous amino acid sequences. They consist of a Gla domain made up of 11–12 glutamic acid residues, 4 epidermal growth factor-like repeat regions, and 1 sex hormone-binding globulin-like domain [26].

The activation of the TAM receptor family relies on the existence of PtdSer [4]. They utilise γ-carboxylation at the γ-position [27]. The carboxylate sequence interacts with PtdSer via binding. Cellular apoptosis is defined by a depletion of intracellular ATP, resulting in an elevation of cytosolic Ca^2+^ levels. This activates the TAM receptors to recognise the PtdSer on the external surface of decaying cells. The "eat me" signal facilitates the identification and elimination of apoptotic cells by macrophages and other phagocytes. PtdSer externalisation is an essential early stage in the cell death process. It serves as a stimulus for macrophages to recognise and consume apoptotic cells. This signal is essential for the initiation of the immune response. Gas6 and Pros1 bind to PtdSer, resulting in the activation of TAM receptors and the start of downstream signalling pathways that regulate immune responses [28].

## Kinetic characteristics of TAM receptors in the tumour microenvironment

The TAM receptor family's dynamic characteristics in the tumour microenvironment manifest in their key roles in mediating cell exocytosis, immune cell regulation, secretion of inflammatory factors, and epithelial–mesenchymal transition. The overexpression of TAM receptors is associated with tumour cell growth, metastasis, invasion, and treatment resistance. But because TAM receptors have many different jobs, they don't always play the same part in tumour immunotherapy. Sometimes, they may even work against the tumour [29]. Elevated AXL expression levels have been associated with treatment resistance in a variety of haematological and solid tumours [30, 31]. For example, aberrant AXL expression has been strongly associated with resistance to erlotinib in non-small cell lung cancer (NSCLC) and to sunitinib in renal cell carcinoma [32].

Various blood cancers, including acute lymphoblastic leukaemia and acute myeloid leukaemia (AML), also exhibit elevated MERTK expression [33, 34], with its expression crucial for tumour cells to evade innate immune surveillance. The phagocytosis of macrophages upregulates MERTK expression, thereby promoting the clearance of apoptotic cells [35]. In glioblastoma, blocking MERTK lowers the number of CD206^+^ M2-like macrophages. This, along with radiotherapy, can greatly slow tumour growth and boost the activation of M1-like macrophages, which improves the immune response that causes inflammation [36]. There is evidence that higher levels of the TYRO3 receptor are linked to a number of cancers, such as squamous cell carcinoma, lung cancer, prostate cancer, thyroid cancer, schwannoma, and multiple myeloma [37–39]. Further studies have found that, due to its unique biological activity in regulating bone homeostasis, TYRO3 also plays a role in the development of tumour bone metastases [40].

## The role of TAM receptors in the escape process of tumour immunity

The expression and regulation of members of the TAM receptor family in the tumour microenvironment significantly influence tumour immunity, as evidenced above. These receptors are involved in the formation of tumour immunity, immune escape, and treatment resistance through multiple mechanisms. AXL regulates the immune responses of NK cells and cytotoxic T cells (CTLs) in human tumours [41]. In the TME of glioblastoma, immune-related cells secrete Pros1, which stimulates the proliferation of glioblastoma cells. As a result, this process stimulates the activation of AXL through phosphorylation, leading to the enhanced ability of tumour cells to resist immune-mediated elimination. When AXL is turned on, tumour cells make immune checkpoint molecules. These molecules can stop NK cells from killing cancer cells and help the immune system avoid detection [42, 43]. In AML, turning on AXL raises the levels of BCL-2 and Twist while lowering the levels of TLR inflammatory setting off. This leads to an augmentation of the immune system's ability to avoid detection [44, 45].

There is a strong link between the abnormal expression of AXL and the clinical and pathological features of people with NSCLC, as well as their outlook [9, 46]. AXL interacts with both epidermal growth factor receptor (EGFR) and ERBB3 to maintain the downstream signalling pathway of NSCLC. While the current PD-1/PD-L1 medications have demonstrated efficacy in the treatment of NSCLC, this pathway serves as the primary mechanism via which NSCLC tumour cells evade immune system detection [47]. Some more research has shown that MERTK, AXL, and other TAM receptors may help the immune system work better by using natural processes inside tumour cells, like making more PD-L1 appear on tumour cells [48]. Previous studies have demonstrated that MERTK stimulates the transcription of PD-L1 in cells undergoing programmed cell death. This process regulates MERTK-induced cell death and immune equilibrium, ultimately promoting tumour progression [49]. Additional research has yielded intriguing discoveries, indicating that PtdSer enhances the efficiency of PD-L1 signalling in T cells and thus verifying the existence of a PtdSer-TAM-PD-L1 axis in breast cancer. The PI3K/Akt signalling pathway has a role in enabling cancers to evade the immune system and become resistant to chemotherapy [48].

In TME, the presence of MERTK in tumour cells may further enhance their independence from growth factors, enable the advancement of the cell cycle, and stimulate tumour growth. This, in turn, increases the ability of tumour cells to spread and move. Furthermore, it can increase the production of anti-inflammatory cytokines by activating M2 macrophages, which helps create an immune-suppressing environment around the tumour [50].

In addition to macrophages, MERTK also affects the function of other immune cells in the TME, including myeloid-derived suppressor cells (MDSCs) and NK cells, which play a crucial role in evading the immune response against tumours. MDSCs are a group of cells with the ability to suppress the immune system. Tumour cells gather in the TME and use a number of methods to stop the body's immune system from attacking the tumour, which weakens anti-tumour immunity [51]. MERTK is involved in regulating the growth, viability, and function of MDSCs, which aids in the evasion of the immune system through malignancies. For example, turning on MERTK can make MDSCs even better at suppressing the immune system by making immune-suppressing molecules like PD-L1 more abundant. PD-L1 binds to PD-1 receptors on T cells, which hinders their activity and promotes the survival of tumour cells [52]. MERTK inhibitors function by impeding MERTK signalling, which results in a reduction in the immunosuppressive capacity of MDSCs. Consequently, this enables the reestablishment of T cell anti-tumour function [53]. This demonstrates that MERTK has the ability to control the PD-1 and PD-L1 pathways in the TME, thereby influencing the local immune system [54]. It has a function in regulating the activity of these cells, which are essential for the immune response.

TYRO3 exerts its influence on DCs through multiple methods. The activation of TYRO3 has a big impact on the maturation of DCs, which in turn changes their ability to gather, process, and present antigens. This may result in insufficient stimulation of T cells, which could weaken the immune response against tumour cells [55]. In addition, TYRO3 can control the movement of DCs from the TME to lymphoid organs, which in turn affects how T cells are stimulated and how many of them there are. In the TME, TYRO3 facilitates the transformation of DCs into a phenotype that suppresses the immune response. TYRO3 accomplishes this by increasing the synthesis of anti-inflammatory cytokines and inhibiting the release of proinflammatory cytokines. Tumours use this method to evade the immune system [56]. TYRO3 activation induces the polarisation of tumour-associated macrophages towards an anti-inflammatory M2 phenotype. Anti-inflammatory cytokines, like IL-10 and TGF-β, are released by polarised macrophages. These cytokines stop T cells from activating and multiplying, which helps cancers get away from the immune system.

In conclusion, the TAM receptor family has the ability of tumour immune escape. Similarly, the TAM receptor family enhances the anti-tumour activity of immune cells such as macrophages and NK cells. This mechanism provides us with the inspiration for the development of targeted anti-tumour drugs by regulating the expression of the TAM receptor family through targeting the TAM receptor family.

## Manipulating the immunological response of the TAM receptor family in the tumour microenvironment

The expression of AXL receptors enhances the phagocytosis process that macrophages carry out, leading to an augmented elimination of tumour cells. This context describes a regulatory mechanism that enhances the efficacy of macrophages by boosting their capacity to eradicate tumour cells [57]. Addition, the activation of the AXL receptor induces the release of cytokines, including TNF-α and IL-6, from macrophages. Cytokines have a vital function in controlling the immune response, specifically by improving the body's capacity to combat malignancies [58]. Macrophages possess the capacity to undergo differentiation into two distinct types: M1, characterised by its anti-tumourigenic properties, and M2, characterised by its protumourigenic properties. Signals from the immediate microenvironment influence the differentiation process. When AXL receptors are activated, they stop the growth of M1 macrophages, which means that fewer inflammatory cytokines are made. The activation of AXL can hinder the production of specific genes typically present in M1-type macrophages, such as iNOS and TNF-α, through the PI3K/Akt signalling pathway. It is possible for AXL to stop the proinflammatory M1-type macrophage state and improve the function of NK cells. It does this by stopping antigen-presenting cells from stopping TLR responses and increasing the activation of NK cells [13, 54]. AXL also has a regulatory role in the formation of NK cells, which are crucial for the surveillance and eradication of tumour cells. When AXL receptors are turned on, they can make NK cells more active by either making them multiply or changing their metabolism to make them more active [59].

Activation of MERTK can maintain immunological tolerance and regulate the proliferation of effector cells. Under normal physiological conditions, MERTK regulates the elimination of dead cells to prevent excessive inflammation, or it inhibits the activation of NF-κb to reduce the release of TNF-α, thereby reducing inflammation [60, 61]. Also, MERTK can attach to PtdSer on the outside of dying cells, which causes phagocytosis to happen [62]. However, under the unique circumstances of the TME, tumour cells may employ MERTK to impede the body's immune response against the tumour [53].

The research has demonstrated that inhibiting MERTK reduces the presence of PD-L1 in the TME, thereby limiting tumour cells' ability to elude the immune system [33].Various tumour cells predominantly express PD-L1 on their surface, while immune cells like DCs and macrophages commonly detect PD-L2. When PD-L1 and PD-L2 bind to the PD-1 receptor, it inhibits the activation and expansion of T cells, which reduces the immune response against tumour cells [33, 39, 40].

**Fig. 1:**
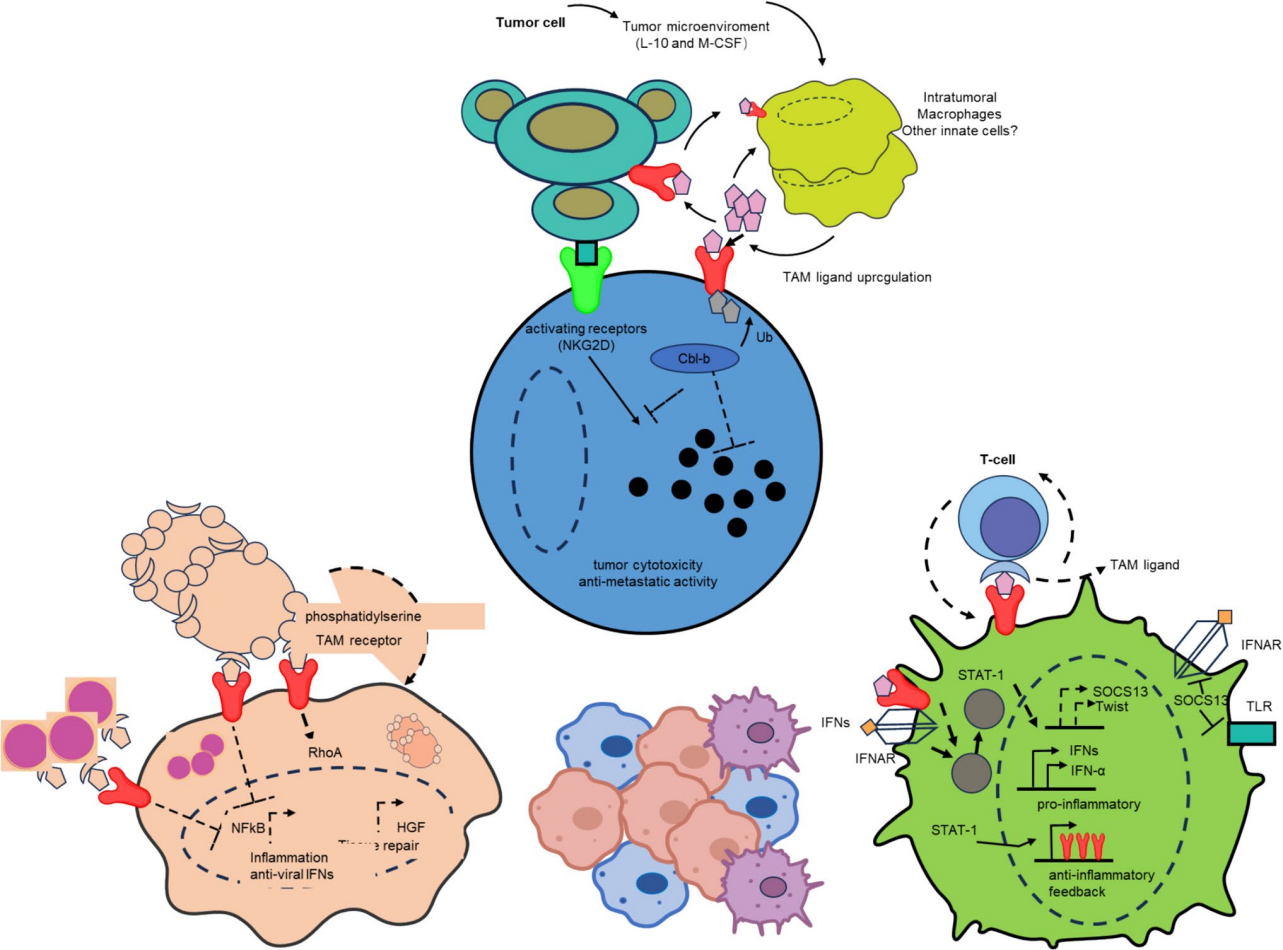
The role of the TAM receptor family in the tumour microenvironment and immune evasion. To help tumour cells grow and stay alive, TAM tumour-associated macrophages release substances like Il-10 and M-CSF into the area around the tumour. At the same time, the activation of TYRO3, AXL, MERTK, and other RTKs on TAM promotes the expression of TAM ligands, forming a positive feedback loop that enhances TAM activity. Macrophages and NK cells are involved in clearing these cells, reducing inflammation, promoting tissue repair by recognising "eat me" signals on the surface of apoptotic cells, and maintaining microenvironmental stability. Furthermore, viral infection in the TME may activate the IFN-αR and Stat-1 signalling pathways, producing type I IFNs, which not only contribute to resistance against viruses but also increase the killing power and antigen-presenting cell function of NK cells and improve the immune surveillance of tumour cells. The anti-virus response can also influence the behaviour of tumour cells by regulating signal molecules like RhoA and NF-κb, thereby influencing the progression of tumours

DCs, NK cells, mononuclear cells, and macrophages all contain TYRO3 [63–66]. Turning on TYRO3 helps NK cells grow and keep fighting tumours [67]. Furthermore, it has the ability to enhance the generation of IFN-γ in NK cells. IFN-γ enhances the activation, proliferation, and differentiation of T cells and NK cells; hence, it augments their capacity to recognise and eradicate tumour cells [68]. IFN-γ makes it easier for tumour cells to make major histocompatibility complex I molecules, which makes antigens show up better. This facilitates the immune system's ability to identify and eradicate tumour cells (Fig. 1).

## Signalling mechanisms of the TAM receptor family

**S**tudies have shown that activated TAM signalling enhances various processes in human malignancies, such as proliferation, migration, invasion, tumour cell survival, angiogenesis, and resistance to chemotherapy drugs [15, 69, 70]. It is crucial to clarify the precise function of AXL in stopping the immunological response. This is a key prerequisite for understanding how to regulate TAM expression and improve the body's anti-tumour immunity.

AXL binds to Gas6 more strongly than TYRO3 or MERTK [58]. Gas6 acts as a binding molecule for TAM receptors. The Gas6-AXL axis is crucial in the process of developing resistance to drugs and evading the immune system [21, 71]. Prior research has demonstrated that increased levels of hMENA, Gas6, and AXL gene expression traits are associated with unfavourable outcomes in pancreatic ductal adenocarcinoma and NSCLC [72]. The Gas6/AX researchers have found that the Gas6/AXL signal makes more immunosuppressant chemokines, such as G-CSF, IL-3, IL-4, IL-6, IL-12_p70, TGF-β, and TNF-α. Research has demonstrated that the Gas6/AXL signalling pathway plays a crucial role in promoting the survival, proliferation, mobility, and invasion of tumour cells [73]. Studies have shown that activation of AXL promotes tumour-associated macrophage polarisation to Type M2, which in turn contributes to tumour growth, enabling tumours to evade the immune system attack [74]. Simultaneously, it diminishes the abundance of CD4⁺ and CD8⁺ T cells within the tumour [75]. T cells and DCs are drawn to the tumour site, but they also facilitate the recruitment of macrophages, neutrophils, and MDSCs [73]. Gas6-mediated mechanisms partially enhance the macrophage polarisation process [76].

The activation of TAM receptors initiates the PI3K/Akt signalling pathway, which plays a critical role in cell proliferation, survival, differentiation, and migration. These pathways increase the ability of tumour cells to survive, grow, and fight apoptosis [77, 78]. Activation of AXL leads to tyrosine residue phosphorylation and strengthens its association with signalling molecules, such as the PI3K p85 subunit and PLC-γ [79]. When Akt is activated, it adds a phosphate group to NF-κb. This makes more of the proteins Bcl-2 and Bcl-xL, which stop cells from dying. This creates a positive feedback loop that enhances the signal for cell survival [80]. Further studies showed that the Gas6/AXL signalling pathway triggers the activation of PI3K/AKT, stimulating the production of anti-apoptotic proteins and thereby inhibiting caspase-3 activity and impeding apoptosis [81]. In addition, it can stimulate ERK and Akt signalling pathways, which affect the proliferation, metastasis, and survival of tumour cells [15, 82].

Inhibiting AXL not only affects tumour cells but also directly affects the immune system, increasing the ability of immune cells to destroy tumour cells. It is important to clarify the exact function of AXL in blocking immune responses. AXL is linked to negative feedback loops of DC and NK cells. Interrupting these loops can boost immune responses in the area around a tumour. Similarly, one theory is that TAM signalling in mice may impair NK cell responsiveness via ubiquitin ligase Cbl-b. Therefore, studies have demonstrated that blocking TAM receptors like AXL enhances NK cell function by reducing the metastatic burden in animal models [83, 84]. In addition, blocking the AXL ligand Gas6 signalling also makes NK cells work harder, which shows how important this pathway is in mouse tumours [85]. However, whether AXL exists in fully developed human NK cells remains controversial. According to one study, AXL mRNA expression ceased at day 10 after the differentiation of CD34^+^ human pluripotent stem cells from NK cells [86]. TYRO3 and AXL share many similarities in signalling. For example, they are both capable of self-dimerisation and phosphorylation through enhanced expression in the absence of ligand binding. Furthermore, TYRO3 is able to respond to Gas6 activation and interacts with the p85 subunit of PI3K, an interaction that is critical for signalling. Recent advances in gene expression microarray analysis of multiple myeloma involving B-lymphoid stem cells have demonstrated the upregulation of TYRO3 expression levels [87]. Similarly, samples from patients with primary malignant plasma cell leukaemia and multiple myeloma also showed the transcriptional activity of TYRO3 mRNA [87]. These findings reveal that TYRO3 and AXL may play an important role in tumour development through interactions with core signalling molecules, such as PI3K.

The activation of MERTK plays an important role in cell signalling, similar to many other RTKs, which trigger a range of classical RTK downstream signalling, including ERK12 and Akt. MERTK signalling in macrophages promotes the synthesis of inflammation resolution mediators by suppressing CaMKII activity; these kinases play key roles in promoting cell invasion, migration, angiogenesis, survival, chemical resistance, and metastasis [50]. Studies have shown that the role of MERTK in NF-κb signalling is twofold: in tumour cells, MERTK activates NF-κb to enhance survival signalling, whereas in myeloid immune cells, including macrophages, it inhibits NF-κb, which in turn inhibits the proinflammatory cytokine response. Also, turning on MERTK doesn't just work through the normal RTK signalling pathway; it also turns on unusual signals in epithelial cells. This impacts MERTK macrophages and helps melanoma grow and become resistant to immunotherapy by activating AhR-ALKAL1. For example, activation of MERTK can lead to Akt-dependent upregulation of PD-L1 on tumour cells [48]. Notably, MERTK may work with other TAM family members, such as AXL. The study by Davra et al. provides an example of this, finding that in both an AXL-driven cancer cell line (E0771) and a MERTK global knockout murine model, independently reducing tumour burden and increasing overall survival led to a tumour-specific T cell response [62]. The same study observed an additive individual benefit when MERTK knockout mice combined with AXL knockout E0771 cells [62].

It is worth noting that TYRO3 and MERTK, despite their structural similarity to AXL, can enhance cell survival through the PI3K/Akt signalling pathway [15]. However, Pros1, as a ligand, is incapable of activating AXL, which highlights the distinctive signalling specificity of TAM receptor members [88].

**Fig. 2:**
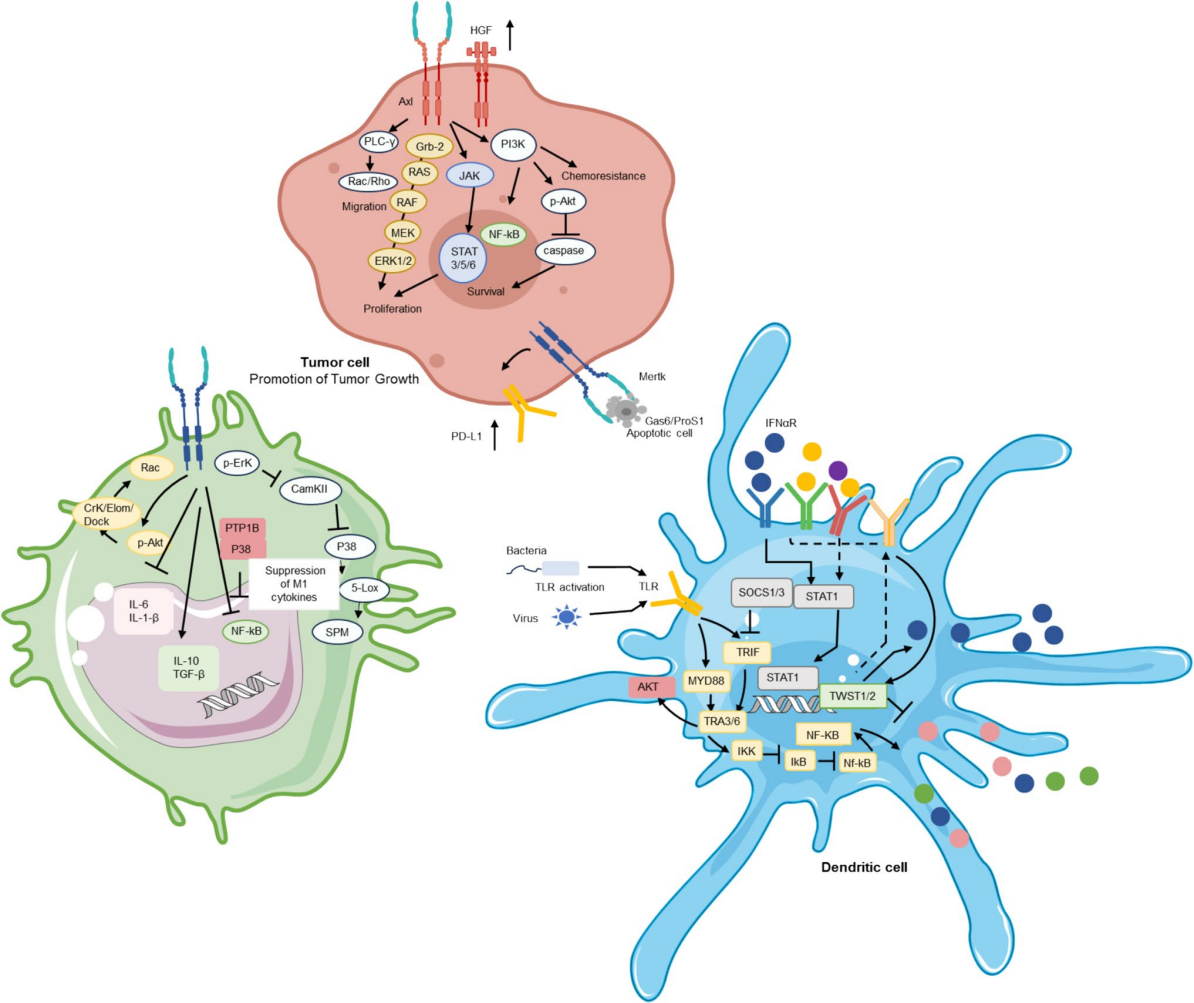
TAM receptor family in tumour microenvironment: driving oncogenesis. In the TME, there is a complex network of cells, including tumour cells, immune cells (such as macrophages and DCs), i.e. infiltrating T cells, and apoptotic cells. In this context, activation of the TAM (TYRO3, AXL, and MERTK) receptor family plays a key role in tumour development and immune escape. Downstream signalling of these receptors activates multiple signalling pathways, such as Jak/Stat, PI3K/p-Akt, and PLC-γ, that contribute to tumour cell proliferation, survival, and drug resistance, which are characteristics of cancer. Activation of the TAM receptor is also associated with increased expression of PD-L1—a signalling molecule that inhibits the adaptive immune system—on tumour cells. In addition, TAM receptors signal inflammation inhibition by inhibiting NF-κb signalling, releasing immunosuppressive cytokines, and promoting the transformation of macrophages from proinflammatory M1 to immunosuppressive M2. The formation of the MERTK-PTP1B-p38 complex, where MERTK activation inhibits p38, contributes to this shift by altering the activity of transcription factors and reducing the expression of type M1 cytokines. TAM receptors also mediate SOCS-dependent suppression of TLR signalling in DCs. TLR activates the expression of NF-κb and proinflammatory cytokine molecules, such as IFN-α, after recognising pathogen-associated molecular patterns. IFN-α induces the expression of AXL through the STAT1 pathway, and AXL interacts with the IFN-α/β receptor (IFN-αR)-STAT1 signalling to promote the expression of SOCS1 and SOCS3. TAM signalling also blocks TLR-mediated inflammation and DC antigen presentations to T cells

The study of the TAM downstream signalling pathway will establish a foundation for future research on targeted therapy of tumour immunity and the optimisation of therapeutic hemes (Fig. 2). According to different targets, different immunosuppressants are used to treat the tumour, and at the same time, personalised special therapeutic schemes or combined therapeutic schemes are made, which provides a brand-new prospect for the development of tumour immunotherapy.

## TAM receptors: new targets and therapeutic options for tumour immunotherapy

Currently, there is proof that the excessive production of AXL is responsible for the development of resistance to chemotherapy in multiple tumour models [89, 90]. The AXL function is associated with resistance to PI3Kα inhibitors. Inhibiting AXL enhances the susceptibility of tumour cells to PI3Kα inhibitors via R428 [90]. This indicates that specifically inhibiting AXL could potentially be effective in overcoming resistance to specific inhibitors. Clinical trials are currently underway to assess the safety and efficacy of TAM receptor inhibitors in tumour treatment.

While TAM receptor inhibitors have demonstrated encouraging outcomes in immunodeficient mice [91], it is imperative to do research in immunocompetent animals to evaluate their potential adverse effects. For instance, the removal of AXL and MERTK genes resulted in a higher occurrence of inflammatory colon cancer in mice [92]. This underscores the need to thoroughly assess the potential negative consequences of TAM receptor inhibitors in clinical settings. Researchers are investigating the use of AXL inhibitors in combination with immune checkpoint inhibitors (ICIs) as a strategy to overcome treatment resistance. Researchers have linked AXL receptors to worse prognosis and treatment resistance in various types of tumours [93]. By focusing on the TAM receptor, namely AXLs, it is possible to improve the effectiveness of current immunotherapies. This is because AXLs are connected to various aspects of the tumour microenvironment, such as immunocytosis, endothelial cell and cell signalling, and communication among tumour cells [69].

Studies have linked elevated expression of TAM receptors in tumour cells to the facilitation of cell survival, proliferation, and migration [94, 95], suggesting that treatments targeting these receptors may have notable efficacy in specific patient groups. When developing personalised treatment plans, it is important to consider the patient's genetic makeup, tumour features, and level of TAM receptor expression (Table 1). Several types of human tumours frequently overexpress or abnormally activate TAM receptors [1, 96]. Customised therapeutic approaches may involve the use of TAM receptor inhibitors in conjunction with other therapeutic drugs, such as EGFR tyrosine kinase inhibitors or immunotherapy [97, 98] (Table 2). This integrated therapeutic approach seeks to not only reduce tumour resistance but also enhance treatment effectiveness. We must tailor customised treatment techniques to the specific conditions of patients, taking into account the changes in TAM receptor expression levels and activity status observed in tumours from various individuals, to ensure optimal efficacy. Furthermore, personalised therapy should also prioritise the possible adverse effects of TAM receptor inhibitors and undergo rigorous evaluation in clinical settings. Through a thorough evaluation of TAM receptor expression and activity, medical professionals can customise a therapy strategy for each patient to optimise therapeutic effectiveness and avoid negative side effects.

**Table 1:** Targeting the TAM receptor for modulation of tumour immunity

Name	Target	Indication	Reference
Tyrosine kinase inhibitors			
Dasatinib	Src family kinases	Chronic myeloid leukaemia, acute lymphoblastic leukaemia, and other potential haematological malignancies	[99]
Bosutinib	Src/Abl tyrosine kinase inhibitor	Chronic myeloid leukaemia, Chronic phase	[100]
Gilteritinib	Highly specific FMS-like tyrosine kinase 3/AXL inhibitor	Relapsed/refractory acute myeloid leukaemia	[101]
Nilotinib	BCR-ABL tyrosine kinase in chronic myeloid leukaemia	Chronic myeloid leukaemia	[102]
Cabozantinib	MET, AXL, VEGFR2 (anti-vascular endothelial growth factor receptor)	Advanced renal cell carcinoma, advanced urothelial carcinoma	[103]
Cabozantinib	AXL (as well as multiple tyrosine kinases including MET)	EGFR-mutant NSCLC	[97]
XL184 (Cabozantinib)	AXL RTK	XL184 (Cabozantinib) for NSCLC	[103]
Erlotinib	EGFR	NSCLC	[32]
BYL719	PI3Kα (Phosphoinositide-3-kinase alpha)	Head and neck squamous cell carcinoma, oesophageal squamous cell carcinoma	[90]
AXL inhibitors			
Bemcentinib (formerly BGB324 or R428), UNC2025, MRX2843, UNC1666	AXL RTK (for Bemcentinib); MERTK (for UNC2025, MRX2843, UNC1666)	Bemcentinib—various solid tumours including brain cancer, triple-negative breast cancer, acute myeloid leukaemia; MERTK inhibitors—AML, ALL, melanoma	[6]
R428	AXL RTK	Mainly breast cancer, potential synergy with other tumour treatments	[7]
R428 (BGB324)	AXL RTK	R428 (BGB324) for acute myeloid leukaemia	[2]
LDC1267	TAM receptor family (TYRO3, AXL, MERTK)	Various tumour models including melanoma and breast cancer	[84]
MERTK inhibitors			
Anti-MERTK antibody	MERTK (MERTK proto-oncogene tyrosine kinase)	MC38 syngeneic murine colon adenocarcinoma cancers and E0771 murine model of triple-negative breast cancer	[53]
Multi-target tyrosine kinase inhibitor			
TP-0903	Multi-kinase inhibitor targeting AXL/AURKA	Neuroblastoma	[10]
DS-1205b	Selective AXL kinase inhibitor	NSCLC	[104]
UNC569, UNC1062	MERTK (MERTK RTK)	Various tumours including leukaemia, NSCLC, glioblastoma, melanoma, prostate cancer, breast cancer, colon cancer, gastric cancer, pituitary adenomas, and rhabdomyosarcomas	[11]
UNC1062	MERTK kinase, associated with various human tumours including acute lymphoblastic leukaemia, acute myeloid leukaemia, NSCLC, and glioblastoma	Acute lymphoblastic leukaemia, acute myeloid leukaemia, NSCLC, glioblastoma	[12]
Bosutinib, Cabozantinib, MGCD265, ASLAN002	Bosutinib—SRC/ABL, AXL; Cabozantinib—VEGFR, MET, FLT3, c-KIT, AXL; MGCD265—MET, AXL; ASLAN002—RON, AURKA, FLT3, MET, AXL	Bosutinib—philadelphia chromosome-positive chronic myeloid leukaemia; Cabozantinib—medullary thyroid cancer, renal cell carcinoma, NSCLC with specific mutations or increased MET or AXL activity	[105]
Other specific target inhibitors			
Venetoclax	Bcl-2 inhibitor	Chronic lymphocytic leukaemia	[106]
MP470	AXL (also blocks other tyrosine kinases at the same concentration)	Gastrointestinal stromal cancers, potentially other drug-resistant cancers	[107]
Warfarin	Cbl-b/TAM receptor in NK cells for anti-metastatic activity	Inhibiting tumour metastasis in mouse models	[84]
Gefitinib, Osimertinib	EGFR-TKIs; AXL RTK	NSCLC	[98]

**Table 2:** Combination therapy with TAM receptor inhibitors

Combination therapy	Target	Indication	Reference
Bemcentinib and pembrolizumab	AXL RTK	NSCLC	[108]
Sitravatinib and nivolumab	TAM receptor family (TYRO3, AXL, MERTK)	Oral cancer	[109]
BGB324 (R428) and cytarabine	AXL RTK	AML	[110]
R428 and cisplatin/paclitaxel	AXL RTK	Lung cancer	[111]
AXL inhibitor and JNJ28312141	CSF-1R, AXL	Melanoma	[112]
Sitravatinib (MGCD516) and nivolumab	TAM receptor family, VEGFR2, MET, RET, KIT	NSCLC	[113]
R428 and BYL719	AXL RTK	Squamous cell carcinoma of the head and neck, squamous cell carcinoma of the oesophagus	[90]
BGB324 and pembrolizumab	AXL RTK	Triple negative breast cancer and adenocarcinoma	[114]
MP470 and docetaxel	c-Kit, AXL	Mesothelioma of the gastrointestinal tract	[115]
Cabozantinib and osimertinib	AXL RTK	NSCLC	[97]

## The challenges and opportunities

Overall, TAM receptors have important effects on various aspects of tumour cell behaviour, including proliferation, tolerance to chemotherapy, migration, and immune evasion. Furthermore, they play a crucial role in the elimination of apoptotic cells, the regulation of inflammatory responses, and the function of immune cells. They play a key role in tumour development by promoting tumour cell growth, survival, migration, and epithelial–mesenchymal transition [15]. System dynamics studies have revealed a close relationship between overexpression or aberrant activation of TAM receptors and tumourigenesis in humans. We discovered that activating the AXL receptor in tumour cells can make them more resistant to the immune system. This is done by changing how NK cells and CTLs respond to the immune system and increasing the production of immune checkpoint molecules like PD-L1. These molecules help tumour cells avoid being detected by the immune system. When it comes to tumour immune escape, the MERTK receptor's job in killing tumour cells and controlling PD-L1 expression are both very important. The TYRO3 receptor is involved in the immune escape mechanism of tumours by affecting the function of DCs and then regulating the activation and immune responses of T cells. In addition, the TAM receptor family plays a dual role in tumour immune escape and anti-tumour immunity and can inhibit tumour development. Early specific mechanisms include influencing macrophage differentiation, regulating phagocytosis by macrophages, enhancing the function of NK cells, and regulating immune distress and inflammatory responses. Discussions on signalling pathways have revealed specific mechanisms underlying the bidirectional regulatory effects of the TAM receptor family on tumours, of which PI3K/Akt and Gas6/AXL are two particularly critical signals. We found that the Gas6-AXL signalling pathway plays a key role in drug resistance and immune escape. Through the above discussion, we clearly identified the TAM receptor family in the tumour immune process as playing a role. In this context, we talk in detail about how TAM receptor inhibitors can be used in personalised cancer treatment plans. To make these plans even more effective, we also talk about how TAM receptor inhibitors work when combined with other therapeutic drugs. Specifically, mixing TAM receptor inhibitors with ICIs like cetrazumab and the anti-PD-1 drug nivolumab offers a new way to deal with tumours that are resistant to drugs and could have a strong therapeutic effect. These findings provide new insights into tumour immunotherapy and lay the foundation for future clinical applications and drug developments [116].

However, there are many obstacles in the way of this research. Firstly, despite the potential therapeutic targets of AXL, MERTK, and TYRO3, their overexpression is associated with therapeutic resistance, indicating the need for more comprehensive studies to overcome this resistance. Second, because TAM receptors can do different things, they don't always play the same role in tumour immunotherapy. Sometimes, they can even work against tumours, which makes it harder to come up with a good treatment plan. In addition, specific regulation of immunosuppressive tumour-associated macrophages to promote anti-tumour immune responses remains an important challenge without interfering with macrophage function in healthy tissues. Our study has not been able to fully assess the potential adverse effects in the clinical setting, which is a shortcoming.

Future studies will require more in-depth studies to understand the specific roles of TAM receptors in different tumour types and how to specifically target these receptors to avoid adverse effects on normal cells. In the future, the application of nano-drug delivery systems may become an important method to solve the problem of drug resistance. We expect strategies like the development of new small-molecule chemical inhibitors and monoclonal antibodies, along with the use of gene-editing techniques, to enhance the effectiveness of immunotherapy. These approaches can achieve this goal by directly blocking TAM receptor signalling or altering the tumour's immune environment.

## Supplementary Information

Below is the link to the electronic supplementary material.

Supplementary file1 (DOCX 126 kb)

## Data Availability

No datasets were generated or analysed during the current study.
