# Supplementary material for: Navigating TAM receptor dynamics in tumour immunotherapy
Source: Cancer Immunol Immunother. 2025 Mar 15;74(5):146. doi: 10.1007/s00262-024-03879-z (PMC11910493; doi:10.1007/s00262-024-03879-z)
Supplement: Supplementary file 1 — Supplementary file1 (DOCX 126 kb) [file 262_2024_3879_MOESM1_ESM.docx]

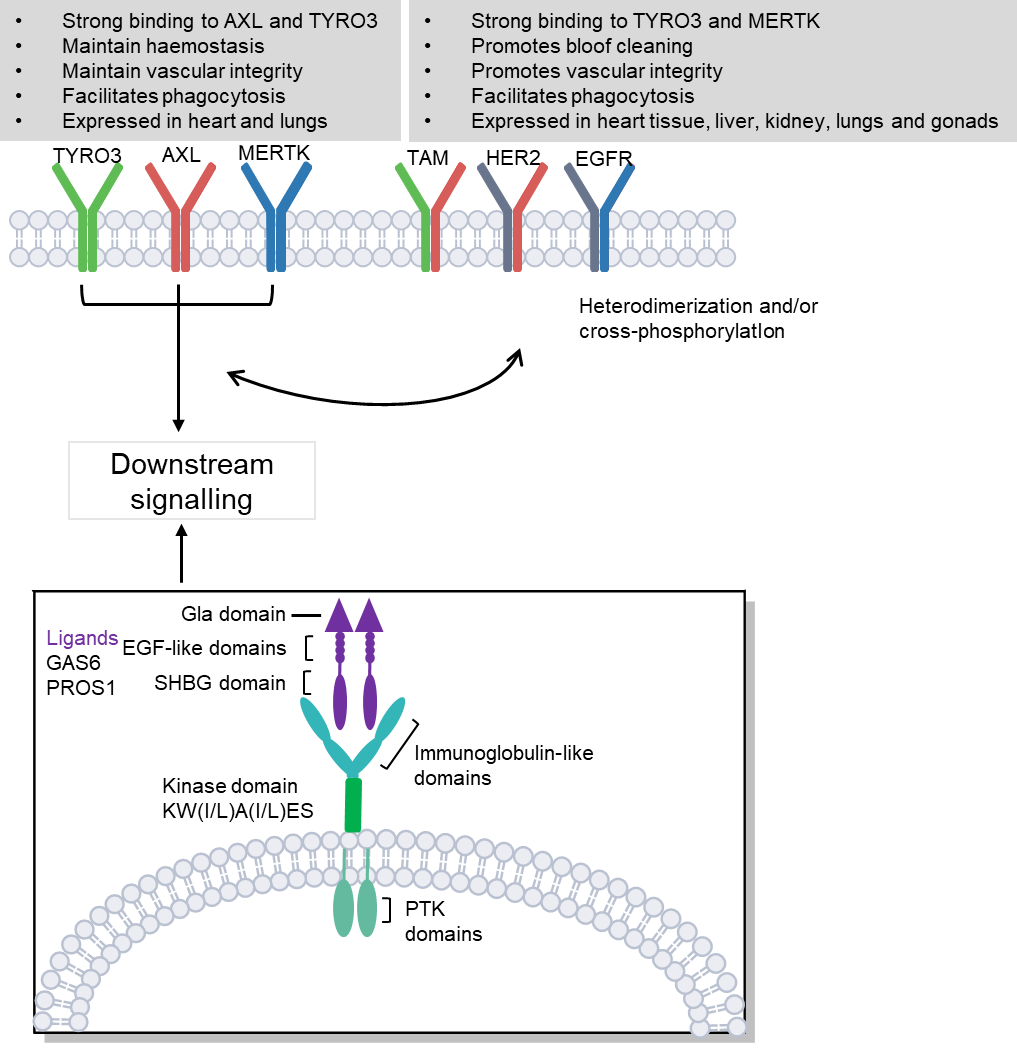


Fig. 1 TAM receptor family domain structure and ligand binding mechanism. The TAM family of RTKs TYRO3, AXL, and MERTK have a similar domain arrangement. In the extracellular part, these receptors all have two immunoglobulin (Ig)-like domains at the N-terminus. Immediately adjacent to the transmembrane region are two domains similar to fibronectin. Usually, for these receptors to work, their Gas6 or vitamin K-dependent Pros1 secreted ligands need to bind to PtdSer on nearby cells. In some cases, high concentrations of PtdSer can also effectively activate the TAM RTKs. Gas6 can bind to and activate all three TAM receptors, while Pros 1 specifically binds to and activates TYRO3 and MERTK
